# Age- and sex-specific care cascades to detect gaps in the care of children with tuberculosis in Bangladesh: a cohort study

**DOI:** 10.7189/jogh.15.04024

**Published:** 2025-01-24

**Authors:** Daniel Ramirez, Amanda Brumwell, Md Mahfuzur Rahman, Farzana Hossain, Suchitra Kulkarni, Amyn A Malik, Jeffrey I Campbell, Brittney J van de Water, Md Kamruzzaman Kamul, Md Toufiq Rahman, Hamidah Hussain, Jacob Creswell, Tapash Roy, Meredith B Brooks

**Affiliations:** 1 Boston University School of Public Health, Boston, Massachusetts, USA; 2 University of Washington, Seattle, Washington, USA; 3 Interactive Research and Development Bangladesh (IRD Bangladesh), Dhaka, Bangladesh; 4 Boston Medical Center, Boston, Massachusetts, USA; 5 IRD Global, Singapore; 6 O’Donnell School of Public Health, UT Southwestern Medical Center, Dallas, Texas, USA; 7 Boston College, Chestnut Hill, Massachusetts, USA; 8 Bangladesh Shishu Hospital and Institute, Dhaka, Bangladesh; 9 Innovations and Grants, Stop TB Partnership, Geneva, Switzerland

## Abstract

**Background:**

Programmatic interventions to increase the detection of children with tuberculosis (TB) are rarely evaluated to understand age- and sex-specific completion rates. We applied modified TB screening and treatment cascade frameworks to assess indicators of effective implementation by age and sex of a TB screening program for children (zero to 14 years) in Bangladesh.

**Methods:**

We implemented an intensified screening program for paediatric TB detection in 119 health care facilities (2018–21). We followed systematic verbal screening by referral for full evaluation for children who reported symptoms or contact history with a patient with TB. Further, we linked children to treatment if diagnosed and followed for outcomes. We calculated the percentage of children, by age and sex, progressing through each step of the care cascade and compared the frequency of step completion by sex using χ^2^ tests.

**Results:**

In total, we screened 552 182 males and 461 419 females for TB. 2.8% of males and 2.6% of females screened positive (*P* < 0.001). 74.2% of males and 73.9% of females underwent appropriate evaluation (*P* = 0.560). 10.3% of males and 11.5% of females were diagnosed with TB (*P* = 0.008). 100% of children initiated treatment, and 97.6% of males and 97.1% of females achieved a successful treatment outcome (*P* = 0.428). The percent of children screening positive on verbal screen, who were clinically evaluated for TB, and who were diagnosed with TB generally increased with age, with some variability throughout (ranges: 1.2–9.1%, 59.8–88.5%, 6.5–21.9%, respectively).

**Conclusions:**

The largest gap observed for both sexes and among all ages was children who were not appropriately evaluated for TB despite screening positive. In our research, we highlight the value of identifying gaps in paediatric TB care to inform innovative, age- and sex-tailored interventions to improve future care in children.

Despite advancements in reducing the incidence of tuberculosis (TB) in recent years, the TB epidemic continues to be characterised by inequalities both in the burden of disease and in the delivery of care [1–3]. Person-centred care is a path forward for advancing improvements in equitable care delivery for vulnerable populations [4,5]. However, implementation of person-centred care requires programmatic acknowledgement of the preferences of people most affected by TB through identification of the most appropriate screening and diagnostic tools, understanding barriers and facilitators to screening and testing, recognising community- and facility-based strategies that are valued and determining effective communication strategies and awareness of ongoing efforts [6]. Children are a uniquely vulnerable population who may not be able to voice their specific preferences, as their medical care often relies on a caregiver. However, evidence suggests that TB care models can be tailored to improve care and management for children with and at risk of TB [7].

Children are a high-priority population for TB programs due to their heightened vulnerability to disease and death, as well as challenges in diagnosing and delivering high-quality TB care. Annually, over one million children and adolescents are estimated to fall sick with TB, and over a quarter million die from the disease [1]. Despite being a high-priority population, only 40% of children estimated to have TB are diagnosed and reported. Furthermore, children less than five years of age are missed more often than children of other age groups [8]. These challenges lead to 96% of TB-related deaths occurring in children who never receive treatment [9]. The World Health Organization (WHO) End TB Strategy has set targets for treatment success rates at >90%. However, child cohorts with TB in low- and middle-income countries have not met this target in recent years [10,11].

Care delivery for TB is often differentiated by the patient’s age, with pre-specified age cut-offs determining clinical practices. However, suggestive age cut-offs can introduce several critical challenges in providing adequate care [12]. Programmatically, children are often aggregated as a singular patient population – under 15 years of age – making it difficult to identify suboptimal care or barriers that are specific to certain vulnerable paediatric subgroups [7]. Clinically, children of different ages are at varying levels of risk of developing active disease and severe forms of TB. Their presentation of symptoms also changes with age [13,14]. Further, as children age, their social networks broaden, and they become capable of communicating more effectively about their own health, losing full reliance on their caregivers. Studies have been consistent in finding that, in cohorts of children aged <15 years, younger children are more likely to be diagnosed with TB and experience worse treatment outcomes at a higher frequency [10,15–17]. Contradictory to this, a study in Bangladesh found that 95% of children diagnosed with TB were in the 10–14-year old age group [18].

Sex-related TB disparities are also observed often. Of note, while sex may contribute to biological risk due to differences in immunological protection, perhaps stronger determinants of risk are gender roles and gendered expectations [19]. Adult males tend to experience a greater burden of disease, present for care at later stages of disease, face work-related financial and physical barriers to care, and experience a difference in immunological protection compared to adult females [20,21]. Adult females can experience reduced access to diagnosis and care due to societally restricted autonomy and financial dependence, household stigma, and reduced health literacy [21]. However, gender-related TB vulnerabilities manifest differently for people of different ages. For example, in Bangladesh, more TB is diagnosed among female children than among male children, which is contrary to epidemiological patterns in adult populations [18]. The risk of children being lost to follow-up has been associated with being female in other settings [22]. It has been found that male children are at higher risk of TB disease and have worse treatment outcomes than their female counterparts [10,19]. However, some studies have shown no difference in treatment outcomes between male and female children [23]. Data on gender-differentiated risk of TB or TB treatment outcomes in paediatric populations is sparse, highlighting the need for further investigation into gender disparities in burden, care, and surveillance [24].

The construction and analysis of TB care cascades is an informative method for assessing the programmatic effectiveness of various care delivery approaches [4,25]. Stratified analysis of care cascades on key patient characteristics, such as age and sex, can identify inequities in care delivery that would otherwise go unnoticed [23]. Conducting age- and sex-stratified cascades of care may enable TB programs to develop more actionable, tailored solutions to close identified gaps in care and ultimately optimise programmatic operations.

In recent years, the Bangladesh National Tuberculosis Program has placed greater priority on the efficient identification of people with TB, particularly among children. However, critical gaps remain in paediatric TB evaluation and diagnosis due partly to the absence of active case finding in facilities, care fragmentation between public and private sector services, and incomplete coverage of rapid and highly sensitive diagnostic tools [26,27]. Moreover, Bangladesh serves as a setting of interest for investigating disparities in care due to prior studies identifying contradictory findings from existing literature, particularly noting that the majority of children diagnosed with TB were female and in the age group of 10–14 years [18]. We constructed a sub-analysis of an intensified tuberculosis screening initiative aimed at increasing the detection of children with TB in Bangladesh. We aimed to identify age- and sex-specific gaps in paediatric TB screening, evaluation, and treatment to inform age- and sex-tailored strategies for future programmatic optimisation.

## METHODS

### Study setting

Between November 2018 and September 2021, Interactive Research and Development, Bangladesh (IRD Bangladesh) conducted an intensified TB screening initiative under the TB REACH Initiative (Wave 6) in Mymensingh Division, Bangladesh, to increase the detection of children with TB. This prospective cohort initially focused on three public and 37 private health facilities between 2018–19. Following this, the program expanded to 38 public and 41 private health facilities between 2019–21. Healthcare personnel at participating facilities received specialised training to verbally screen all children attending paediatric outpatient and inpatient departments using an integrated decision support system.

### Data collection and measurements

We implemented a multifaceted strategy to identify paediatric TB, including systematic screening in clinics, household contact tracing, and the establishment of a source patient identification system within households of identified child TB patients. We extended invitations to contacts and made efforts to facilitate their visits to health facilities. In instances where attendance was not possible, project staff conducted home visits. Verbal screenings were systematically conducted during these interactions, accompanied by complimentary TB tests.

We extended the data collection process to both public and private health care facilities. Health workers utilised an Android phone-based electronic screening tool for systematic verbal screenings of children in paediatric outpatient and inpatient departments. Individuals who screened positive underwent clinical assessment and diagnostic testing at no cost. During screening, we collected clinical and demographic data, encompassing variables such as sex, age, weight, symptoms, family medical history, and details of the hospital where screening and TB treatment were administered. We deployed a modified TB cascade indicator framework [28,29], comprising six steps along the TB care continuum for children.

#### Step 1: screened for TB

We identified children as screened for TB once a health worker verbally screened the children in the paediatric outpatient departments of participating facilities using the Android phone-based electronic screening tool. The screening process involved health workers inquiring for: 1) a cough lasting two weeks or longer; 2) symptoms such as fever, fever duration, night sweats, and inappropriate weight loss (or failure to thrive); 3) glandular swelling; or 4) contact with someone with TB in the last two years.

#### Step 2: positive TB screen

We classified children as having a positive screen if they responded that they had contact with someone with TB in the last two years, had a cough greater than two weeks, had glandular swelling, and/or when two or more of the symptoms were present (fever lasting two or more weeks, night sweats, or weight loss). Anyone with a positive screen was deemed presumptive for TB.

#### Step 3: evaluated for TB

We classified children as having been evaluated for TB if they tested positive during the screening process and subsequently underwent: 1) a comprehensive assessment by a medical officer; and either 2) clinical evaluation with the chest x-ray, computed tomography scan, histopathology, lymph node examination, or abdominal ultrasound; or 3) TB diagnostic tests, including smear microscopy or GeneXpert mycobacterium TB complex/resistance to rifampin assay.

#### Step 4: diagnosed with TB

Children were diagnosed with TB if a TB medical officer made an official final diagnosis based on either bacteriologic confirmation of disease, or a combination of clinical, laboratory, radiological, and/or histopathological test results. Children were classified as having either bacteriologically confirmed or clinically diagnosed TB disease.

#### Step 5: started TB treatment

We referred children who started TB treatment to the National TB Control Program and had dates of treatment initiation recorded.

#### Step 6: successful TB treatment outcome

We defined a successful TB treatment outcome as either achieving a bacteriologic cure or completing TB treatment as per the national guidelines [30].

### Analysis

We calculated the percentage of children completing each step in the care cascade by dividing the number of children who completed each step by the number eligible for each step (per the number completing the prior step) (**Table 1**). We did this separately by sex (males and females) and age (each year of a child’s age at screening from zero to 14). We also calculated the percentages of completion of each cascade step for males and females separately within each year of age. We then assessed the difference in frequency of completion of each step by gender by calculating the χ^2^ test statistics and associated *P*-values.

**Table 1:** Sex-specific care cascade results for children aged zero to14 y (2018–21)*

Care cascade step	Male†	Female†	*P*-value
Step 1: screened	552 182 (100)	461 419 (100)	NA
Step 2: screened positive	15 262 (2.8)	12 018 (2.6)	<0.001
Step 3: evaluated	11 327 (74.2)	8882 (73.9)	0.560
Step 4: diagnosed	1169 (10.3)	1020 (11.5)	0.008
*Bacteriologic confirmation*	85 (7.3)	56 (5.5)	0.090
Step 5: initiate treatment	1169 (100)	1020 (100)	NA
Step 6: successful outcome	1141 (97.6)	990 (97.1)	0.428

NA – not applicable

*Presented as n (%) unless specified otherwise.

**†**Male (n = 552 182; 54.5%), female (n = 461 419; 45.5%).

If we observed large gaps in completion at any cascade step, we compared characteristics across children who did and did not complete that step using χ^2^ tests to understand gaps in completion. Characteristics explored included sex, age, and clinical symptoms reported at screening, including the presence of cough, fever, weight loss, glandular swelling, night sweats, poor appetite, recent contact with someone with TB, and the presence of a Bacille Calmette-Guerin (BCG) scar. We completed analyses using SAS, version 9.4 (SAS Institute Inc, Cary, NC, USA).

### Ethics approval and consent to participate

The Institutional Review Board of BRAC James P Grant School of Public Health, BRAC University, Bangladesh, reviewed and approved the original study protocol (approval number 2019-037-ER). We obtained verbal informed consent from all children’s guardians and from children over the age of seven. The Boston University Institutional Review Board (document number H-43034) determined that secondary analysis of this de-identified data was non-human subjects research.

## RESULTS

Between 2018–21, 552 182 male children and 461 419 female children aged zero to 14 years underwent verbal TB screening (**Table 1**). The denominator of how many children attended the facilities could not be determined, so the percentage of those screened could not be calculated. Among those who did get screened, 15 262 (2.8%) males and 12 018 (2.6%) females screened positive (*P* < 0.001) and were eligible for further evaluation for TB disease. 11 327 (74.2%) males and 8882 (73.9%) females were then evaluated for TB disease (*P* = 0.560). A subsequent 1169 (10.3%) males and 1020 (11.5%) females were diagnosed with TB (*P* = 0.008), with 85 (7.3%) males and 56 (5.5%) females who were diagnosed with TB having bacteriologic confirmation of disease (*P* = 0.090). All male and female children with TB disease were linked to care and initiated TB disease treatment, and 1141 (97.6%) males and 990 (97.1%) females experienced a successful treatment outcome (*P* = 0.428).

**Figure 1:**
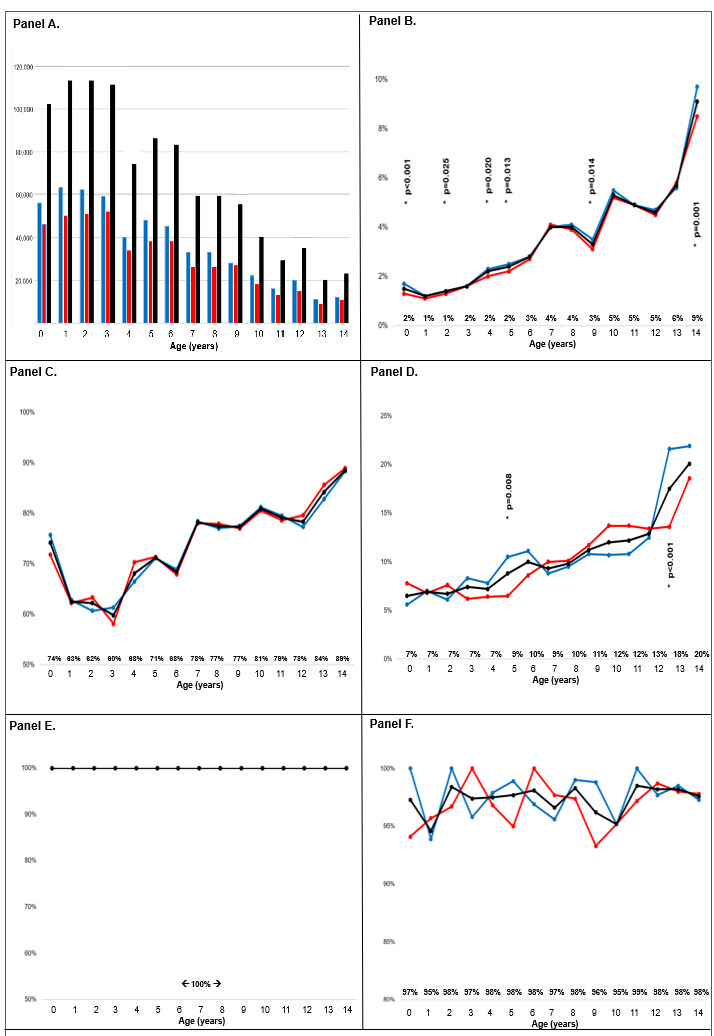
Progression of children throughout each step of the TB care cascade, stratified by age and sex. **Panel A.** Children screened for TB, by age and sex (n = 1 007 387). **Panel B.** Percent of children screening positive for TB, out of those who were screened, by age and sex (n = 27 055; 2.7%). **Panel C.** Percent of children evaluated for TB, out of those who screened positive, by age and sex (n = 20 107; 74.3%). **Panel D.** Percent of children diagnosed with TB, out of those who were clinically evaluated, by age and sex (n = 2189; 10.9%). **Panel E.** Percent of children who started TB treatment out of those who were diagnosed with TB, by age and sex (n = 2189; 100%). **Panel F.** Percent of children with a successful outcome of those who started treatment, by age and sex (n = 2131; 97.4%). The black line represents males and females combined. The red line represents females, and the blue line represents males. Percentages reported above x-axis are for the black lines (males and females combined). *P*-values indicate significant differences in care observed across genders (*P* < 0.05). TB – tuberculosis.

Age was not reported for 6361 (0.6%) children. Children aged <5 years made up 51.3% (n = 516 356) of the total children screened, while children aged five to 9 years old accounted for 34.2% (n = 344 377), and those aged 10–14 years old accounted for only 14.7% (n = 146 654). As age increased, the number of children screened decreased (**Figure 1**, Panel A; Table S1 in the **Online Supplementary Document**). While there was some variability in the percentage of children who screened positive for TB as age increased, we generally observed an increasing trend, with a low percentage of 1.2% for children one year old to a high percentage of 9.1% for children 14 years old) (**Figure 1**, Panel B; Table S1 in the **Online Supplementary Document**). Significantly more males aged zero (*P* < 0.001), two (*P* = 0.025), four (*P* = 0.020), five (*P* = 0.013), nine (*P* = 0.014), and 14 years (*P* = 0.001) screened positive than their female counterparts of the same age.

Of those who screened positive, the percentage who were subsequently appropriately evaluated for TB was variable (**Figure 1**, Panel C). 74.2% of children aged <1 year were evaluated, then the percentage dipped to only 59.8% for three-year-olds and then rose until peak completion of evaluation (88.5% for 14-year-olds). No sex-specific differences by age were observed for TB evaluation. The percentage diagnosed with TB was lowest in children aged <1 year (6.5%), rose to 8.8% in children aged five years old, 12.0% in children 10 years old, and 20.1% in children 14 years old (**Figure 1**, Panel D). Significantly more males ages five (*P* = 0.008) and 13 (*P* < 0.001) years were diagnosed with TB than their female counterparts of the same ages.

All children initiated treatment (**Figure 1**, Panel E), and successful treatment outcomes were similar across all ages, with a low of 94.6% among children one-year-old and a high of 98.5% among children 11 years old (**Figure 1**, Panel F). No sex-specific gaps by age were observed in the frequency of children with a successful TB treatment outcome.

Upon closer examination, the largest gap in cascade step completion across all ages and sexes was being evaluated for TB after screening positive. We identified several characteristics associated with completing this step. A smaller percentage of children who completed an evaluation were aged <5 years compared to children who did not complete an evaluation (25.1% *vs.* 38.6%; *P* < 0.001) (**Table 2**). Additionally, a higher percentage of children who reported cough, fever, weight loss, swelling, night sweats, poor appetite, and had the BCG vaccine completed an evaluation compared to those who did not (*P* < 0.001) (**Table 3**). A lower percentage of children who had recent contact with someone with TB completed an evaluation compared to those who did not complete an evaluation (42.5% *vs.* 44.6%; *P* = 0.003).

**Table 2:** Age and sex of children presumed to have tuberculosis disease who did and did not complete a tuberculosis evaluation*

Child characteristics	Children who completed an evaluation (n = 20 209)	Children who did not complete an evaluation (n = 7017)	*P*-value
Male sex	11 327 (56.1)	3935 (55.7)	0.560
Age†			<0.001
*0*	1146 (5.7)	399 (5.7)	
*1*	813 (4.0)	487 (7.0)	
*2*	953 (4.7)	580 (8.4)	
*3*	1045 (5.2)	702 (10.1)	
*4*	1100 (5.5)	516 (7.4)	
*5*	1469 (7.3)	594 (8.6)	
*6*	1579 (7.9)	729 (10.5)	
*7*	1884 (9.4)	525 (7.6)	
*8*	1812 (9.0)	529 (7.6)	
*9*	1413 (7.0)	415 (6.0)	
*10*	1742 (8.7)	412 (5.9)	
*11*	1126 (5.6)	298 (4.3)	
*12*	1255 (6.2)	348 (5.0)	
*13*	941 (4.7)	177 (2.6)	
*14*	1829 (9.1)	237 (3.4)	

*Presented as n (%) unless specified otherwise.

†Children who completed an evaluation (n = 20 107); children who did not complete an evaluation (n = 6948).

**Table 3:** Clinical characteristics and symptoms of children presumed to have tuberculosis disease who did and did not complete a tuberculosis evaluation*

Clinical characteristics	Children who completed an evaluation	Children who did not complete an evaluation	*P*-value
Cough	16 632/20 092 (81.5)	415/773 (53.7)	<0.001
Fever	16 886/20 090 (84.1)	387/774 (50.0)	<0.001
Weight loss	13 512/20 084 (67.3)	3725/6983 (53.3)	<0.001
Swelling	3755/19 999 (18.8)	1452/6985 (20.8)	<0.001
Night sweats	7454/20 089 (37.1)	110/774 (14.2)	<0.001
Poor appetite	12 691/20 076 (63.2)	354/774 (45.7)	<0.001
Presence of BCG scar	19 676/20 081 (98.0)	741/774 (95.7)	<0.001
Recent contact with TB	8530/20 055 (42.5)	3137/7033 (44.6)	0.003

BCG – Bacille Calmette-Guerin, TB – tuberculosis

*Presented as n (%) unless specified otherwise.

## DISCUSSION

We found notable differences by sex and age in completing different steps of the TB care cascade for paediatric TB management. Investigating sex-specific gaps in the diagnosis of paediatric TB is a critical endeavour, as it addresses potential disparities in health care outcomes among children. We revealed sex-specific gaps, indicating disparities in screening and diagnosis rates between males and females. Males represented a greater proportion of the population receiving screening (54.5% male *vs.* 45.5% female). This proportion surpasses the estimated proportion of the male paediatric population in our setting (50.5% in 2024) [31,32]. Notably, more males screened positive, while more females were diagnosed with TB. This finding corresponds with results from other studies, which highlight sex-related nuances in accessing and benefiting from TB services [5,33–37]. This finding is converse of what is often observed in adults, in which males are diagnosed more often than women but, based on prevalence survey results, are missed more often by the health systems because they are not seeking care [24]. The intricate interplay of sociocultural, economic, and health care system factors explored in our research sheds light on the complexity of these disparities. Societal norms and expectations, access to health care services, health care provider biases, symptom recognition and reporting, education, and awareness levels are all critical aspects influencing these gaps. Qualitative research methods, including interviews and surveys with caregivers, health care providers, and community members, can help unravel the complexities of these disparities. Developing gender-responsive interventions can help address these barriers for children as well as adults [38,39].

Age-related factors play a significant role in barriers to TB detection, diagnosis, treatment, and management. Specifically, adolescents face TB-related stigma and challenges accessing adolescent-friendly health services, leading to underdiagnosis, while younger children encounter diagnostic uncertainties due to difficulties in obtaining appropriate samples [5,33]. These trends are consistent with challenges highlighted in studies exploring childhood TB in low- and middle-income countries [36]. These detailed numerical insights underscore the need for targeted interventions to improve screening rates, especially in older age groups, while recognising and addressing specific gender-based disparities in diagnosis. The uniformity our study identified in treatment outcomes emphasises the effectiveness of the treatment phase in paediatric TB care.

In our analysis, we revealed age- and sex-specific trends in differential screening and diagnosis rates. Understanding these interactions is crucial for tailoring interventions to increase care. Our findings provide valuable insights that can inform the development of targeted strategies, aligning with the need for more comprehensive methods and estimates in paediatric TB care [36]. Recognising age and sex dynamics allows for designing context-specific interventions, optimising TB care delivery and improving health equity for vulnerable populations. These dynamics may be salient to other initiatives working to understand and resolve disparities in care delivery in other settings where age and sex affect access to and the quality of TB care.

Similar to previous studies, we observed that the largest gap in cascade step completion – for all ages and both sexes – was among children who screened positive for TB but did not complete an evaluation [40,41]. This gap poses a challenge because it impedes the accurate identification and management of TB patients, leading to missed diagnoses and potential ongoing transmission. One possible reason for this is inequitable access to evaluation processes. Studies have shown that many patients may not be able to access health services due to financial and time constraints [42–44]. Healthcare visits often result in time off from work and lost wages for parents and guardians and time off from school for children. Previous work has also highlighted the difficulties in diagnosing TB in children, particularly in obtaining appropriate samples and dealing with limitations in diagnostic sensitivity. The consistent identification of this gap across different studies underscores the urgent need for improved strategies in evaluating children presumed to have TB [45,46]. However, it is also important to acknowledge the potential complexities surrounding the non-completion of the evaluation for certain children. These complexities may include things such as lack of protocol adherence, recent use of TB preventive therapy and perception of redundancy in re-evaluation. Further research to better understand the complexities of non-completion of cascade steps can lead to future tailored interventions to effectively close these gaps in care.

One limitation of our study is the absence of other patient-level characteristics, including comorbidities or social factors, such as the financial status of the household or the education level of the parent, which could not be collected due to the retrospective nature of the chart review and the programmatic nature of data collection. This limitation hinders a comprehensive understanding of potential comorbidities and social factors that may have had a direct or indirect impact on the child’s health or influenced the care-seeking behaviour of caregivers, possibly contributing to going undiagnosed or unsuccessful treatment outcomes. Additionally, there were numerous different points of contact for screening included in the overall tuberculosis screening intervention, including out-patient and in-patient departments, contact investigations, and public and private facilities. Our study does not differentiate the difference in completion of cascade steps across these different screening entry points, which may hold valuable information regarding where children of different ages and sexes are screened and provided care. Further, we were unable to ascertain the status of TB preventive therapy initiation and completion among children in whom TB disease was ruled out. TB preventive therapy is a critical component of a comprehensive TB elimination strategy; further insights into age- and sex-specific initiation and completion rates among children could identify important gaps in services that should be addressed rapidly. Further, we did not have access to the total number of children attending the facilities, leaving the percentage of children screened for TB unable to be calculated. This limitation impacts our ability to assess the reach of the screening program and its potential biases in who takes part. Despite these challenges, our study stands as a substantial contribution, providing critical insights into the paediatric TB care landscape. Moreover, we identified factors associated with the failure to complete a critical step of the cascade, offering crucial information for tailoring future quality improvement interventions. Further research should delve into health facility- and systems-level factors, such as infrastructure, resources, and personnel, to better understand barriers to a child being appropriately evaluated for TB once identified as high risk for the disease.

Our study has many strengths, including comprehensively incorporating age, sex, and their interaction in conducting cascade analysis for paediatric screening and treatment programs in Bangladesh, building on our previous work from Pakistan [23]. The program’s substantial size and large number of children screened add to the robustness of our study, enabling a detailed analysis of sequential steps in the TB care journey for children. This extensive data set not only serves as a cornerstone to evaluate paediatric TB epidemiology in Bangladesh but also enhances the depth and reliability of our findings.

## CONCLUSIONS

In this comprehensive examination of the paediatric TB care cascade within the context of an intensified TB screening initiative in Bangladesh, we uncovered notable variations by age and gender that demand tailored strategies for improving screening rates, evaluation processes, and diagnosis outcomes, especially in older age groups. A multifaceted approach is essential to bridge the identified gaps in paediatric TB care. This approach should be context-specific and encompass the use of improved diagnostic tools, targeted health care provider training programs, and robust community engagement initiatives. By addressing these aspects, we can enhance the accuracy and efficiency of TB management and contribute significantly to the global efforts aimed at eliminating TB in children. Further research exploring the underlying reasons for these age- and sex-specific trends, and facility level-variability in cascade step completion, is warranted to inform targeted interventions aimed at narrowing diagnostic gaps and ensuring equitable care provision. Specifically, the role of socioeconomic factors to better understand caregiver decision-making and cultural and health system factors that may impact screening and diagnosis practices is important to further explore.

## Additional material


Online Supplementary Document
