# Supplementary material for: Age- and sex-specific care cascades to detect gaps in the care of children with tuberculosis in Bangladesh: a cohort study
Source: J Glob Health. 2025 Jan 24;15:04024. doi: 10.7189/jogh.15.04024 (PMC11758173; doi:10.7189/jogh.15.04024)
Supplement: Online Supplementary Document [file jogh-15-04024-s001.pdf]

**Supplement to: Ramirez D, Brumwell A, Rahman MM, Hossain F, Kulkarni S, Malik AA, Campbell JI, van de Water BJ, Kamrul MK, Rahman MT, Hussain H, Creswell J, Roy T, Brooks MB. Age- and sex-specific care cascades to detect gaps in the care of children with tuberculosis in Bangladesh: a cohort study. J Glob Health. 2025;15:04024.**

**Table S1. Age- and sex-specific completion of each step of the pediatric tuberculosis care cascade**

| Age | **Step1:** Screened N=1007387 | **Step 2:** Screened positive N=27,055 | **Step 3:** Evaluated N=20,107 | **Step 4:** Diagnosed N=2,189 | *Bacteriologic confirmation N=141* | **Step 5:** Initiate treatment N=2,189 | **Step 6:** Successful outcome N=2,131 |
|---|---|---|---|---|---|---|---|
| 0 | 103053 (10.2%) | 1545 (1.5%) | 1146 (74.2%) | 74 (6.5%) | *3 (4.1%)* | 74 (100%) | 72 (97.3%) |
| M | 56580 (54.9%) | 939 (1.7%) | 711 (75.7%) | 40 (5.6%) | *1 (2.5%)* | 40 (100%) | 40 (100%) |
| F | 46473 (45.1%) | 606 (1.3%) P<0.001 | 435 (71.8%) P=0.084 | 34 (7.8%) P=0.143 | *2 (5.9%)* | 34 (100%) | 32 (94.1%) P=0.120 |
| 1 | 113341 (11.3%) | 1300 (1.2%) | 813 (62.5%) | 56 (6.9%) | 1 (1.8%) | 56 (100%) | 53 (94.6%) |
| M | 63163 (55.7%) | 755 (1.2%) | 474 (62.8%) | 33 (7.0%) | 1 (3.0%) | 33 (100%) | 31 (93.9%) |
| F | 50178 (44.3%) | 545 (1.1%) P=0.086 | 339 (62.2%) P=0.831 | 23 (6.8%) P=0.922 | 0 (0%) | 23 (100%) | 22 (95.7%) P=0.779 |
| 2 | 113629 (11.3%) | 1533 (1.4%) | 953 (62.2%) | 64 (6.7%) | 4 (6.3%) | 64 (100%) | 63 (98.4%) |
| M | 62160 (54.7%) | 882 (1.4%) | 558 (63.3%) | 34 (6.1%) | 3 (8.8%) | 34 (100%) | 34 (100%) |
| F | 51469 (45.3%) | 651 (1.3%) P=0.025 | 395 (60.7%) P=0.302 | 30 (7.6%) P=0.362 | 1 (3.3%) | 30 (100%) | 29 (96.7%) P=0.283 |
| 3 | 111502 (11.1%) | 1747 (1.6%) | 1045 (59.8%) | 77 (7.4%) | 6 (7.8%) | 77 (100%) | 75 (97.4%) |
| M | 59749 (53.6%) | 946 (1.6%) | 580 (61.3%) | 48 (8.3%) | 5 (10.4%) | 48 (100%) | 46 (95.8%) |
| F | 51753 (46.4%) | 801 (1.6%) P=0.634 | 465 (58.1%) P=0.166 | 29 (6.2%) P=0.210 | 1 (3.5%) | 29 (100%) | 29 (100%) P=0.265 |
| 4 | 74831 (7.4%) | 1616 (2.2%) | 1100 (68.1%) | 79 (7.2%) | 3 (3.8%) | 79 (100%) | 77 (97.5%) |
| M | 40931 (54.7%) | 930 (2.3%) | 618 (66.5%) | 48 (7.8%) | 3 (6.3%) | 48 (100%) | 47 (97.9%) |
| F | 33900 (45.3%) | 686 (2.0%) P=0.020 | 482 (70.3%) P=0.104 | 31 (6.4%) P=0.395 | 0 (0%) | 31 (100%) | 30 (96.8%) P=0.752 |
| 5 | 86854 (8.6%) | 2063 (2.4%) | 1469 (71.2%) | 129 (8.8%) | 3 (2.3%) | 129 (100%) | 126 (97.7%) |
| M | 48096 (55.4%) | 1198 (2.5%) | 852 (71.1%) | 89 (10.5%) | 3 (3.4%) | 89 (100%) | 88 (98.9%) |
| F | 38758 (44.6%) | 865 (2.2%) P=0.013 | 617 (71.3%) P=0.917 | 40 (6.5%) P=0.008 | 0 (0%) | 40 (100%) | 38 (95.0%) P=0.177 |
| 6 | 83883 (8.3%) | 2308 (2.8%) | 1579 (68.4%) | 158 (10.0%) | 5 (3.2%) | 158 (100%) | 155 (98.1%) |
| M | 45858 (54.7%) | 1278 (2.8%) | 880 (68.9%) | 98 (11.1%) | 5 (5.1%) | 98 (100%) | 95 (96.9%) |

| | | | | | | | |
|---|---|---|---|---|---|---|---|
| F | 38025 (45.3%) | 1030 (2.7%) P=0.491 | 699 (67.9%) P=0.610 | 60 (8.6%) P=0.093 | 0 (0%) | 60 (100%) | 60 (100%) P=0.171 |
| 7 | 59738 (5.9%) | 2409 (4.0%) | 1884 (78.2%) | 176 (9.3%) | 3 (1.7%) | 176 (100%) | 170 (96.6%) |
| M | 33277 (55.7%) | 1321 (4.0%) | 1035 (78.4%) | 91 (8.8%) | 1 (1.1%) | 91 (100%) | 87 (95.6%) |
| F | 26461 (44.3%) | 1088 (4.1%) P=0.381 | 849 (78.0%) P=0.851 | 85 (10.0%) P=0.366 | 2 (2.4%) | 85 (100%) | 83 (97.7%) P=0.456 |
| 8 | 58996 (5.9%) | 2341 (4.0%) | 1812 (77.4%) | 177 (9.8%) | 11 (6.2%) | 177 (100%) | 174 (98.3%) |
| M | 33406 (56.6%) | 1354 (4.1%) | 1043 (77.0%) | 99 (9.5%) | 7 (7.1%) | 99 (100%) | 98 (99.0%) |
| F | 25590 (43.4%) | 987 (3.9%) P=0.226 | 769 (77.9%) P=0.614 | 78 (10.1%) P=0.645 | 4 (5.1%) | 78 (100%) | 76 (97.4%) P=0.427 |
| 9 | 54906 (5.5%) | 1828 (3.3%) | 1413 (77.3%) | 158 (11.2%) | 8 (5.1%) | 158 (100%) | 152 (96.2%) |
| M | 28361 (51.7%) | 996 (3.5%) | 772 (77.5%) | 83 (10.8%) | 6 (7.2%) | 83 (100%) | 82 (98.8%) |
| F | 26545 (48.4%) | 832 (3.1%) P=0.014 | 641 (77.0%) P=0.813 | 75 (11.7%) P=0.573 | 2 (2.7%) | 75 (100%) | 70 (93.3%) P=0.073 |
| 10 | 40320 (4.0%) | 2154 (5.3%) | 1742 (80.9%) | 209 (12.0%) | 12 (5.7%) | 209 (100%) | 199 (95.2%) |
| M | 21920 (54.4%) | 1199 (5.5%) | 973 (81.2%) | 104 (10.7%) | 4 (3.9%) | 104 (100%) | 99 (95.2%) |
| F | 18400 (45.6%) | 955 (5.2%) P=0.214 | 769 (80.5%) P=0.713 | 105 (13.7%) P=0.059 | 8 (7.6%) | 105 (100%) | 100 (95.2%) P=0.988 |
| 11 | 28820 (2.9%) | 1424 (4.9%) | 1126 (79.1%) | 137 (12.2%) | 7 (5.1%) | 137 (100%) | 135 (98.5) |
| M | 15503 (53.8%) | 766 (4.9%) | 609 (79.5%) | 66 (10.8%) | 6 (9.1%) | 66 (100%) | 66 (100%) |
| F | 13317 (46.2%) | 658 (4.9%) P=0.999 | 517 (78.6%) P=0.666 | 71 (13.7%) P=0.139 | 1 (1.4%) | 71 (100%) | 69 (97.2%) P=0.170 |
| 12 | 35034 (3.5%) | 1603 (4.6%) | 1255 (78.3%) | 162 (12.9%) | 10 (6.2%) | 162 (100%) | 159 (98.2%) |
| M | 19588 (55.9%) | 911 (4.7%) | 704 (77.3%) | 88 (12.5%) | 6 (6.8%) | 88 (100%) | 86 (97.7%) |
| F | 15446 (44.1%) | 692 (4.5%) P=0.448 | 551 (79.6%) P=0.259 | 74 (13.4%) P=0.626 | 4 (5.4%) | 74 (100%) | 73 (98.7%) P=0.665 |
| 13 | 19732 (2.0%) | 1118 (5.7%) | 941 (84.2%) | 165 (17.5%) | 16 (9.7%) | 165 (100%) | 162 (98.2) |
| M | 10380 (52.6%) | 576 (5.6%) | 477 (82.8%) | 65 (13.6%) | 6 (9.2%) | 65 (100%) | 64 (98.5%) |
| F | 9352 (47.4%) | 542 (5.8%) P=0.455 | 464 (85.6%) P=0.201 | 100 (21.6%) P=0.001 | 10 (10.0%) | 100 (100%) | 98 (98.0%) P=0.828 |
| 14 | 22748 (2.3%) | 2066 (9.1%) | 1829 (88.5%) | 368 (20.1%) | 49 (13.3%) | 368 (100%) | 359 (97.6%) |
| M | 11517 (50.6%) | 1115 (9.7%) | 984 (88.3%) | 183 (18.6%) | 28 (15.3%) | 183 (100%) | 178 (97.3%) |

| | | | | | | | |
|---|---|---|---|---|---|---|---|
| F | 11231 (49.4%) | 951 (8.5%) P=0.001 | 845 (88.9%) P=0.668 | 185 (21.9%) P=0.080 | 21 (11.4%) | 185 (100%) | 181 (97.8%) P=0.723 |

*Note: 1,007,387 have year of age reported; percentages are calculated by rows; p-values represent the difference in cascade step completion across sex within a specific age (M: male; F: female).*
